# Diagnosis and Surgical Management of Exophytic Suprasellar Pituitary Adenoma Extending Over the Diaphragma Sellae and Mimicking Craniopharyngioma: A Case Report

**DOI:** 10.7759/cureus.10028

**Published:** 2020-08-25

**Authors:** Shohei Noguchi, Kohei Suzuki, Yoshiteru Nakano, Mitsuo Yamaguchi-Okada, Junkoh Yamamoto

**Affiliations:** 1 Neurological Surgery, University of Occupational and Environmental Health, Kitakyusyu, JPN; 2 Hypothalamic and Pituitary Surgery, Toranomon Hospital, Tokyo, JPN

**Keywords:** exophytic pituitary adenoma, silent corticotroph adenoma, endoscopic transsphenidal surgery, fiesta, ultrasonic aspiration, craniopharyngioma, ectopic pituitary adenoma, suprasellar pituitary adenoma

## Abstract

Pituitary adenomas developing from the lateral surface of the pituitary gland are referred to as exophytic pituitary adenomas. When an exophytic pituitary adenoma extends into the suprasellar region, the tumor exhibits an atypical growth pattern that makes it difficult to distinguish it from craniopharyngiomas or other parasellar lesions on MRI.

A 53-year-old woman who presented with general malaise and visual disturbances was diagnosed with a brain tumor. MRI showed a suprasellar tumor presenting as superior lobulation with reticular enhancement and partial calcification. Subsequently, endoscopic transsphenoidal surgery was performed on the patient. The suprasellar tumor was found to originate from the superior surface of the normal pituitary gland and it extended into the supra-diaphragm region. Subtotal tumor resection was achieved, and her clinical symptoms subsequently improved.

Exophytic suprasellar pituitary adenomas (SPAs) are extremely rare and may be mistaken for ectopic SPAs in some cases. Contrast-enhanced fast imaging employing steady-state acquisition (CE-FIESTA) can clearly depict the connection between an exophytic SPA and the normal pituitary gland via a diaphragma sellae defect. During surgery, it was seen that the exophytic SPA and anterior lobe of the pituitary gland connected with each other directly. The tumor originated from the superior surface of the pituitary gland and extended into the supra-diaphragm region. These findings clearly confirmed the difference between exophytic SPAs and ectopic SPAs. In surgical management, an exophytic SPA needs careful consideration for resecting the tumor from encased surrounding structures without vascular and nerve injury. Ultrasonic aspiration devices may be useful for safely resecting the tumor from important structures due to tissue selection.

Exophytic SPAs are distinguished from ectopic SPAs with respect to the direct connection between the tumor and the normal pituitary gland. These findings can be clearly depicted using CE-FIESTA and should be confirmed during surgery. Clinicians should be aware of the risk that exophytic SPA may extend into the supra-diaphragm region and of the difficulties of resecting the tumor encasing surrounding structures in the suprasellar region.

## Introduction

Pituitary adenomas are the most common parasellar lesions that originate in the pars distalis and they frequently occur in sella turcica [1]. They grow on a path of least resistance with the expansion of the sella turcica and extend into the surrounding structures, such as the sphenoid sinus, cavernous sinuses, or the suprasellar region [2]. In some cases, pituitary adenomas present with exophytic growth pattern and are commonly referred to as exophytic pituitary adenomas. Exophytic pituitary adenomas develop from the lateral or inferior surface of the pituitary gland and extend into the cavernous sinus or sphenoid sinus without obvious expansion of the sella turcica [3]. Occasionally, pituitary adenomas develop from the superior surface of the pituitary gland and extend only into the subarachnoid space without enlargement of the sella turcica, similar to the spreading pattern as observed in craniopharyngiomas [4].

In this report, we present a rare case of a craniopharyngioma-like exophytic suprasellar pituitary adenoma (SPA) that was located only in the suprasellar region. This case highlights the importance of studying exophytic SPA for differential diagnoses in parasellar lesions.

## Case presentation

**Figure 1:**
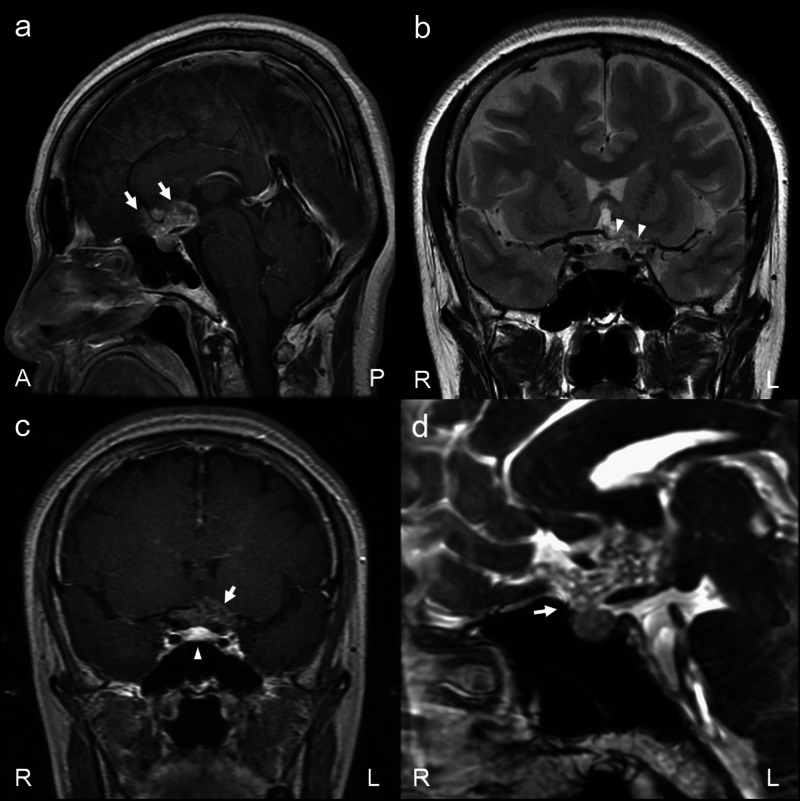
Preoperative MRI findings Sagittal T1-weighted contrast and coronal T2-weighted magnetic resonance images showed a homogeneous intensity mass in the sella turcica and a lobulated reticular enhancement tumor (arrows) encasing optic nerves and cerebral arteries (arrowheads) in the suprasellar region (a, b). Dynamic enhanced MRI showed delayed enhancement of the suprasellar tumor (arrow) compared to that of the normal pituitary gland (arrowhead) (c). Sagittal CE-FIESTA MRI detected a diaphragm defect (arrow) and the tumor extending into the supra-diaphragm region (d) MRI: magnetic resonance imaging; CE-FIESTA: contrast-enhanced fast imaging employing steady-state acquisition

A 53-year-old woman, who complained of general malaise and visual disturbances for the past two months, had been first diagnosed with a brain tumor and then referred to our hospital. On admission, serological examination showed no endocrinopathy [adrenocorticotropic hormone (ACTH): 18.8 pg/ml; cortisol: 9.3 μg/dl; growth hormone: 0.3 ng/ml; insulin-like growth factor 1: 107 ng/ml; prolactin: 8.0 ng/ml; thyroid-stimulating hormone: 2.36 μIU/ml; free T4: 1.68 ng/dl], and normal response for ACTH loading test. Neurological examination showed bitemporal hemianopia, which was confirmed using the Goldmann perimeter. MRI revealed a suprasellar tumor with superior lobulation and reticular gadolinium enhancement (Figure 1A). The tumor encased the anterior cerebral artery (ACA), anterior communicating artery (Acom), perforating branches, and optic chiasm without enlargement of the sella turcica (Figure 1B). Dynamic enhanced MRI showed delayed enhancement of the suprasellar tumor as compared to that of the normal pituitary gland (Figure 1C). In addition, contrast-enhanced fast imaging employing steady-state acquisition (CE-FIESTA) imaging showed that the suprasellar tumor was connected to the normal pituitary gland through the defect of the diaphragm sellae (Figure 1D).

CT scan showed a partial classification of the tumor. Based on these radiological findings, we considered a suprasellar tumor, such as a craniopharyngioma or an ectopic SPA, as a preoperative diagnosis.

**Figure 2:**
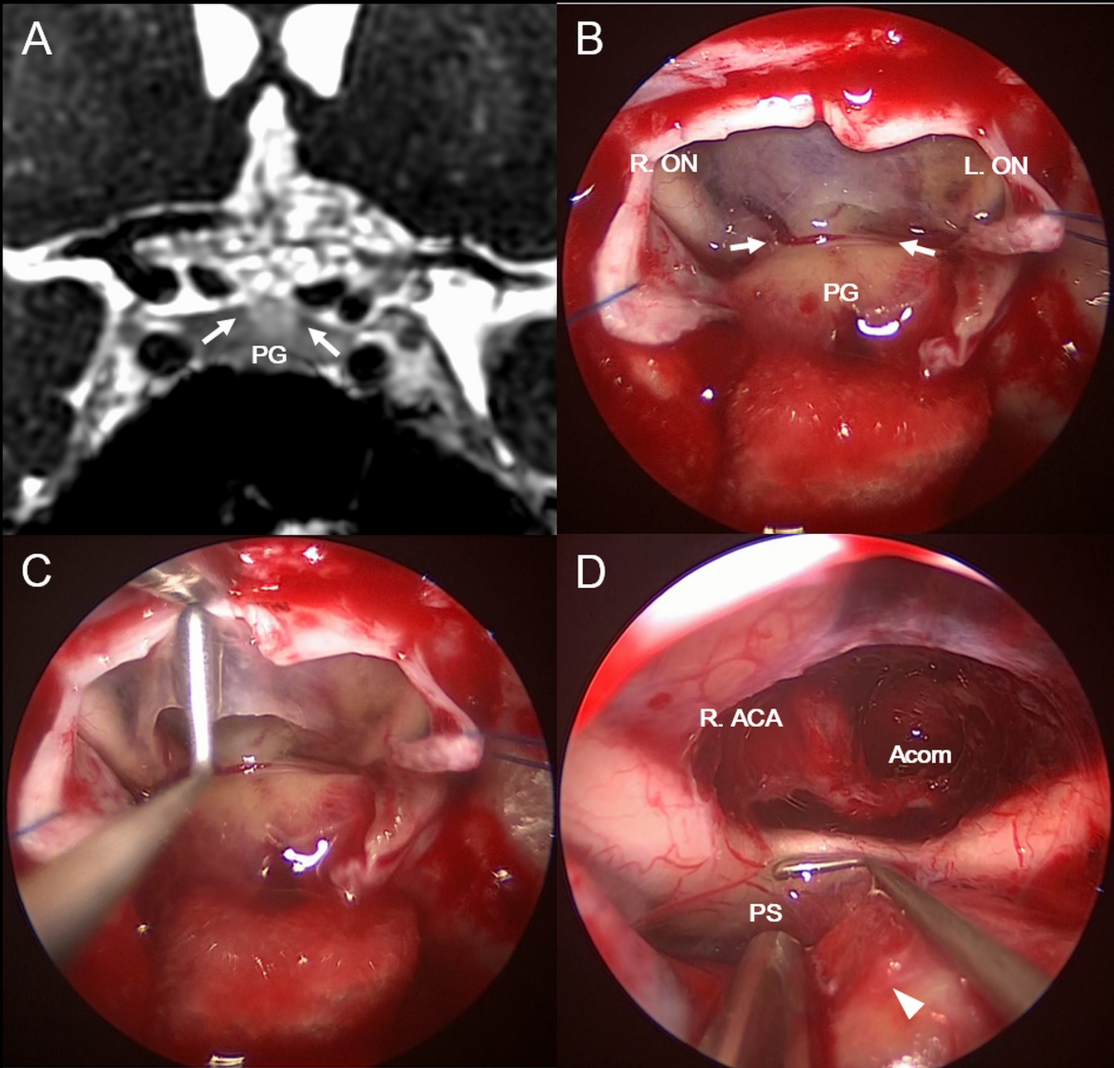
Intraoperative findings The tumor was exposed via the extended endoscopic transsphenoidal approach. Comparison of diaphragm defects (arrows) detected on preoperative coronal CE-FIESTA MRI and surgery (A, B). The tumor arose from the superior surface of the pituitary gland and extended into the supra-diaphragm region through a diaphragm defect (C). The intrasellar pituitary gland had a normal appearance after tumor resection except for a connecting point (arrowhead) with the tumor, and an intact pituitary stalk was observed under the optic chiasm (D) ON: optic nerve; PG: pituitary gland; ACA: anterior cerebral artery; Acom: anterior communicating artery; PS: pituitary stalk; MRI: magnetic resonance imaging; CE-FIESTA: contrast-enhanced fast imaging employing steady-state acquisition

Because the tumor had extended relatively posteriorly, the patient underwent endoscopic transsphenoidal surgery (Figures 2A-2D), in which the dura overlying the planum sphenoidale was opened using the extended transsphenoidal approach.

**Figure 3:**
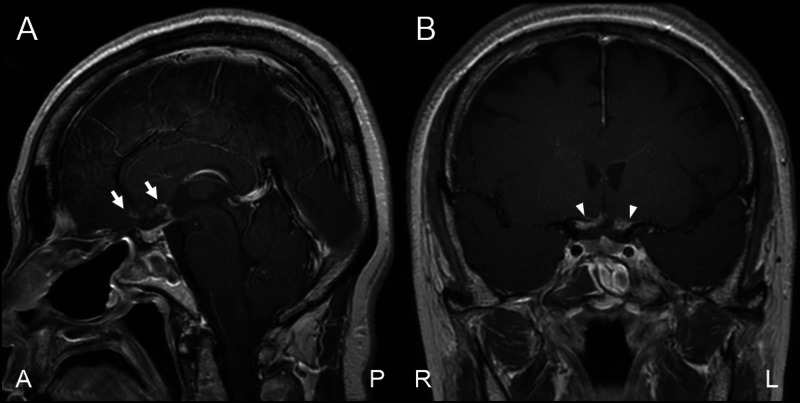
Postoperative MRI findings Sagittal and coronal contrast-enhanced T1-weighted images showed that almost all suprasellar tumors were resected (arrows) except at the lateral region and around the laminar terminalis region (arrowheads) (A, B) MRI: magnetic resonance imaging

The main tumor component was located above the diaphragma sellae, and after the incision of the diaphragma sellae and arachnoid membrane, the tumor was exposed. It originated from the anterior lobe surface of the pituitary gland and extended into the suprasellar subarachnoid space through the diaphragm defect from the attachment. The optic chiasm dislocated inferiorly without adhesion, whereas the ACA, Acom, and their perforators were buried in the tumor. Hence, we carefully resected the tumor using an ultrasonic aspiration device without causing injury to the surrounding structures. The pituitary stalk could be observed behind the dislocated optic chiasm without adhesion to the tumor. The normal pituitary gland without thinning was detected in the sella turcica. Subtotal resection of the tumor was achieved, except around the laminar terminalis (Figures 3A, 3B).

**Figure 4:**
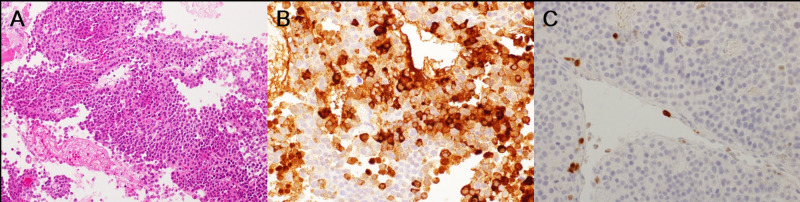
Histological examination findings Histological examination showed diffusely labeled adenoma cells with deposits of hemosiderin (A). Immunohistochemical staining was focally positive for ACTH (B), and the MIB-1 labeling index was approximately 3% (C) ACTH: adrenocorticotropic hormone

Histological examination demonstrated a pituitary adenoma exhibiting focal immunoreactivity for ACTH. The MIB-1 labeling index was approximately 3% (Figures 4A-4C).

Based on the intraoperative findings, the patient was diagnosed with an exophytic SPA. After surgery, the patient’s visual field defect recovered without any other neurological deficits. The postoperative endocrinological test results were also normal. Adjuvant radiotherapy for the residual tumor was performed on account of the high MIB-1 labeling index, at four months after surgery.

## Discussion

An exophytic pituitary adenoma is defined here as a pituitary adenoma that develops from the marginal surface of the pituitary gland rather than inside its structure [3]. The incidence rate of exophytic pituitary adenoma is unclear since only a small number of cases have been reported so far. Previous studies have described that wider diaphragmatic foramina and intrasphenoidal septation are associated with suprasellar extensions [2,5]. According to several reports, an association between exophytic growth and ACTH expression in adenoma cells is also suggested. In patients with ACTH-producing adenomas, microadenomas with diameters of 2 mm or less can be exophytic inside the subarachnoid space, can reach the surface of the anterior lobe, and can invade the cavernous sinus [4]. In the context of multiple endocrine neoplasia type 1, the ACTH-producing adenoma, which most commonly occurs during prepubertal periods, can also be exophytic, growing into the subarachnoid space, or invading the cavernous sinus or wall [6]. Patients with silent corticotroph adenomas, which are clinically nonfunctioning but immunochemically ACTH-positive, have also shown cavernous sinus invasion more frequently as compared to those with gonadotroph adenomas [7]. Although information about transcriptional factors was not obtained in the present case because of limitations in the examination, the adenomas were clinically nonfunctioning but presented with ACTH expression immunochemically. Exophytic SPA may be considered as one variation of the growth pattern of ACTH-producing pituitary adenomas. Exophytic growth from the early stage may lead to extension into surrounding structures without normal pituitary gland thinning.

An exophytic SPA is extremely rare, not clearly defined, and might be reported as ectopic SPA in some cases. In fact, the suprasellar region is one of the most predominant locations for ectopic pituitary adenomas [8]. Although it is difficult to differentiate between exophytic and ectopic SPAs, exophytic SPA should be distinguished as a differential diagnosis of ectopic SPA, as in the present case. Ectopic pituitary adenomas are thought to arise from embryological remnants of the pituitary tissue or from anterior lobe cells attached to the supra-diaphragmatic portion of the pituitary stalk [9]. The most important method used to diagnose suprasellar ectopic pituitary adenoma is to ascertain whether the tumor has a direct connection either to the intrasellar pituitary gland or the pituitary stalk, or to neither of them [10]. A previous study has classified ectopic SPAs into three types: type 1 originating from the anterior pituitary gland, and extending superiorly through the diaphragma sellae; type 2 originating from pars tuberalis; and type 3 originating from residual cells of Rathke’s pouch. Only type 3 is theoretically real ectopic SPA without any direct connection with the pituitary gland [11]. According to this classification, the present case is classified as type 1, which is the rarest subtype. In the present case, the tumor arose from the superior anterior pituitary surface, which was located in the infra-diaphragm region, and it extended via a diaphragm defect into the supra-diaphragm region. The diaphragma sellae did not exist between the pituitary gland and supra-diaphragm tumor, and the tumor was directly connected to the pituitary gland. These findings were confirmed by intraoperative observations. To the best of our knowledge, this is the first reported case of an exophytic SPA, which is classified as ectopic SPA type 1. Our findings were confirmed by the anatomic origin of the superior portion of the anterior pituitary tissue. An ectopic SPA type 1 should therefore be called an exophytic pituitary adenoma, and it should be distinguished from other types of ectopic SPAs accordingly.

Although its characteristics are not well known, both radiological and intraoperative findings are important for the diagnosis of exophytic SPA. Generally, pituitary adenomas exhibit extensions into the surrounding structures with the enlargement of the sella turcica and thinning of the pituitary gland resulting in the so-called snowman-like appearance, as well as homogenous enhancements on MRI [1]. In the present case, the tumor presented with superior lobulation with encasing of the artery and optic chiasm with compression of the third ventricle; however, MRI revealed no dislocation of the normal pituitary gland and no enlargement of the sella turcica. In addition, the tumor presented partial calcification in the solid portion on CT and MRI. These radiological findings suggested a craniopharyngioma, which typically has an ovoid shape or superior lobulation with compression of the third ventricle and a reticular enhancement pattern without the sella turcica enlargement, rather than a typical pituitary adenoma; therefore, it was difficult to distinguish parasellar lesions based only on preoperative radiological findings. On the other hand, preoperative CE-FIESTA detected the diaphragm defect and the SPA connection with the superior surface of the pituitary gland directly. CE-FIESTA has high spatial resolution and can clearly depict the connection between the suprasellar tumor and the pituitary gland via the defect of the diaphragma sellae. Thus, CE-FIESTA is useful for distinguishing between exophytic SPA and other parasellar lesions. In addition, a confirmation of those findings intraoperatively led to the diagnosis of an exophytic SPA. As previously mentioned, a direct connection of SPAs and a normal pituitary gland in the superior surface of anterior lobe trough via a diaphragm defect should be confirmed to distinguish them from other parasellar lesions, including ectopic SPAs. It is important for clinicians to check this using preoperative radiological findings, such as CE-FIESTA imaging, and to confirm during surgery that exophytic SPAs do grow from the superior surface of the pituitary gland.

For the surgical management of SPAs that extend into the suprasellar region, it is important to determine whether the tumor extends into the supra-diaphragm. If the tumor exists in the suprasellar but infra-diaphragm regions, the tumor does not attach to the surrounding structures directly; however, if it extends into the supra-diaphragm region, that is, into the suprasellar subarachnoid space, the tumor may encase the surrounding structures directly. In addition, especially during transsphenoidal surgery, supra-diaphragm tumor resection leads to cerebrospinal fluid leakage, and reconstruction of the anterior skull base or sellar floor becomes essential. In fact, a previous report has described a study in which 12 cases of ectopic pituitary adenoma underwent transsphenoidal surgery, with half of the cases requiring secondary transcranial surgery because of the difficulties in resecting the suprasellar tumor [12]. In the present case, we considered that the tumor extended into the supra-diaphragm region encasing the surrounding structures directly based on MRI findings. We prepared an ultrasonic aspiration device to resect the tumor from the vessels and nerves. Ultrasonic transducers limit the damage to blood vessels and nerves during tumor resection due to tissue selection, which is beneficial for patient prognosis [13]. In cases where the tumor extends into the supra-diaphragm region, these devices are considered safe for use in surgery.

## Conclusions

Exophytic SPAs that develop from the superior surface of the pituitary gland rarely present with atypical development mimicking craniopharyngioma. Radiological and intraoperative findings are important for defining the diagnosis of exophytic SPA. In the case of a supra-diaphragm extension of exophytic pituitary adenoma, the tumor may encase suprasellar surrounding structures directly, and clinicians should be aware of the risks related to supra-diaphragm pituitary adenoma resection to ensure safety in surgical management.
